# Effect of Tobacco Control Policies on Information Seeking for Smoking Cessation in the Netherlands: A Google Trends Study

**DOI:** 10.1371/journal.pone.0148489

**Published:** 2016-02-05

**Authors:** Sigrid A. Troelstra, Jizzo R. Bosdriesz, Michiel R. de Boer, Anton E. Kunst

**Affiliations:** 1 Department of Public Health, Academic Medical Center, University of Amsterdam, Amsterdam, the Netherlands; 2 Department of Health Sciences, Faculty of Earth and Life Sciences, VU University, Amsterdam, the Netherlands; University College London, UNITED KINGDOM

## Abstract

**Background:**

The impact of tobacco control policies on measures of smoking cessation behaviour has often been studied, yet there is little information on their precise magnitude and duration. This study aims to measure the magnitude and timing of the impact of Dutch tobacco control policies on the rate of searching for information on smoking cessation, using Google Trends search query data.

**Methods:**

An interrupted time series analysis was used to examine the effect of two types of policies (smoke-free legislation and reimbursement of smoking cessation support (SCS)) on Google searches for ‘quit smoking’. Google Trends data were seasonally adjusted and analysed using autoregressive integrated moving average (ARIMA) modelling. Multiple effect periods were modelled as dummy variables and analysed simultaneously to examine the magnitude and duration of the effect of each intervention. The same analysis was repeated with Belgian search query data as a control group, since Belgium is the country most comparable to the Netherlands in terms of geography, language, history and culture.

**Results:**

A significant increase in relative search volume (RSV) was found from one to four weeks (21–41%) after the introduction of the smoking ban in restaurants and bars in the Netherlands in 2008. The introduction of SCS reimbursement in 2011 was associated with a significant increase of RSV (16–22%) in the Netherlands after 3 to 52 weeks. The reintroduction of SCS in 2013 was associated with a significant increase of RSV (9–21%) in the Netherlands from 3 to 32 weeks after the intervention. No effects were found in the Belgian control group for the smoking ban and the reintroduction of SCS in 2013, but there was a significant increase in RSV shortly before and after the introduction of SCS in 2011.

**Conclusions:**

These findings suggest that these tobacco control policies have short-term or medium-term effects on the rate of searching for information on smoking cessation, and therefore potentially on smoking cessation rates.

## Introduction

Smoking is the leading cause of preventable deaths in high income countries and is associated with an increased risk of lung cancer, ischemic heart disease, stroke, high blood pressure, oesophagus cancer and chronic obstructive pulmonary disease [1]. In 2012, the prevalence of cigarette smoking in the Netherlands was estimated at 26% [2]. Around 80% of the smokers wanted to quit smoking, 28% attempted to quit smoking in the past year but only 4 to 10% of the smokers trying to stop succeeded in this for at least a year [2].

Effective tobacco control legislation and services are considered the key factor to reduce smoking prevalence and tobacco related diseases [3]. The Netherlands has implemented several tobacco control policies since 2000. In 2003, the Dutch Tobacco Act was amended to include a ban on sales to youth under sixteen and a ban on sponsoring and advertising. Furthermore, major anti-tobacco campaigns were launched on a national level [4]. In 2004, national smoke-free legislation in the workplace was implemented, followed by smoke-free legislation in hospitals and nursing homes in 2005 and a smoke-free legislation in the hospitality industry in 2008 [5]. In 2011, reimbursement of smoking cessation support (SCS) by health insurance was implemented, which was cancelled in 2012 but reintroduced in 2013.

Research on the long-term effects of Dutch tobacco control legislation suggests that introducing smoke-free legislation has increased quit attempts. However, no effect was found on successful quit attempts [5]. A study on causality of smoke-free legislation and smoking cessation suggested that smoke-free legislation could influence smoking cessation through changing attitudes towards smoking cessation and intention to quit smoking [6]. Furthermore, it was stated that the Dutch smoke-free hospitality legislation might have lost its effectiveness due to non-comprehensiveness and unsupportive media attention. However, the magnitude and duration of these effects on short to mid-term outcomes remain unknown. Quit attempts are challenging to measure, since this requires a large study population and often relies on self-reported behaviour. Furthermore, estimating effects of policy on quitting can be challenging due to potential confounding by other concurring events. Therefore, precursors of quit attempts for smoking may be needed to measure with greater precision the relation between the implementation of tobacco control policies and smoking cessation.

Data on smoking cessation-related behaviour could be used to examine the magnitude and duration of the effects of tobacco control policies. Smoke-free legislation can increase smokers’ thinking of smoking cessation by creating growing awareness of the harms of smoking and by reinforcing negative social norms towards smoking. This may produce a growing intention to quit smoking, which may have long-term effects on smoking cessation [7]. It is suggested that in countries with a high level of tobacco control policies, a larger proportion of smokers intends to quit within the next six to twelve months [8]. However, very few large scale studies have been performed on the effect of tobacco control policies on precursors of smoking cessation and cessation-related behaviour [9]. As a result, there is little information on the precise magnitude and duration of the effect of various tobacco control policies [5].

Based on studies on the effect of smoking cessation policies on quit attempts, we hypothesize that the introduction of smoke-free legislation will cause a temporary increase in search queries with a maximum duration of six months [10–14]. Based on findings from two RCTs and a real life study on the effects of reimbursement of SCS, it is expected that the reimbursement of SCS will cause a longer increase in search queries of maximally two years [15–17].

In this article, internet search query data are applied in quasi-experimental design to measure the effect of tobacco control policies on a national level. The aim of this study is to measure the magnitude and duration of the impact of the Dutch smoke-free legislation and reimbursement of smoking cessation support in terms of search query data. In order to assess the validity of our results, we have included Belgium as a control group, since Belgium and the Netherlands share the same language, culture and history and they have a similar smoking prevalence [18].

## Methods

### Ethics statement

In the Netherlands, medical research is governed by the Medical Research Involving Human Subjects Act (‘Wet Medisch-wetenschappelijk Onderzoek met mensen'), which is based on the principles of the declaration of Helsinki. This law only applies if study participants are subjected to any action, treatment or behaviour. Written confirmation from the Medical Ethics Review Committee of the Academic Medical Center that the Medical Research Involving Human Subjects Act was obtained stating that this law does not apply to this study and therefore no official approval was required.

### Data

Internet search query data collected from Google Trends have previously been used to predict influenza epidemics [19], unemployment rates [20] and seasonal depression [21]. Google Trends search query data has recently been used to analyse behavioural patterns like consumer interest in non-cigarette tobacco products [22], the use of electronic cigarettes [23,24] and the relation between tax avoidance and a cigarette tax increase [25,26]. This previously conducted research [19–26] shows Google Trends to be a useful tool to analyse behavioural patterns which are difficult to measure otherwise.

Google Trends data is publicly available and can be downloaded directly from http://www.google.com/trends. Google Trends data are scaled to the average search traffic (the number of searches conducted at a certain point of time) for a selected point in time. The outcome variable, relative search volume (RSV) measures the total number of searches conducted for the selected query compared with the total number of Google searches executed at that point in time. The time period with the highest relative amount of search queries of the chosen term is assigned a value of 100. Other time periods obtain a score relative to 100 [27]. Because of the indexing used, Google Trends has the possibility to compare search queries with other search queries, between geographic regions and within broader periods of time [27,28].

We used Google Adwords (google.com/adwords), to collect the average monthly search volume for various smoking cessation related search queries [26]. Because the Dutch equivalent to ‘quit smoking’ (‘stoppen met roken’) was by far the most used search query (93% for the Netherlands and 90% for Belgium), compared to alternatives like ‘how do I quit smoking’, ‘help quit smoking’ and ‘smoking withdrawal symptoms’, this term was used as outcome in the analysis.

### Analytical design

**Fig 1:**
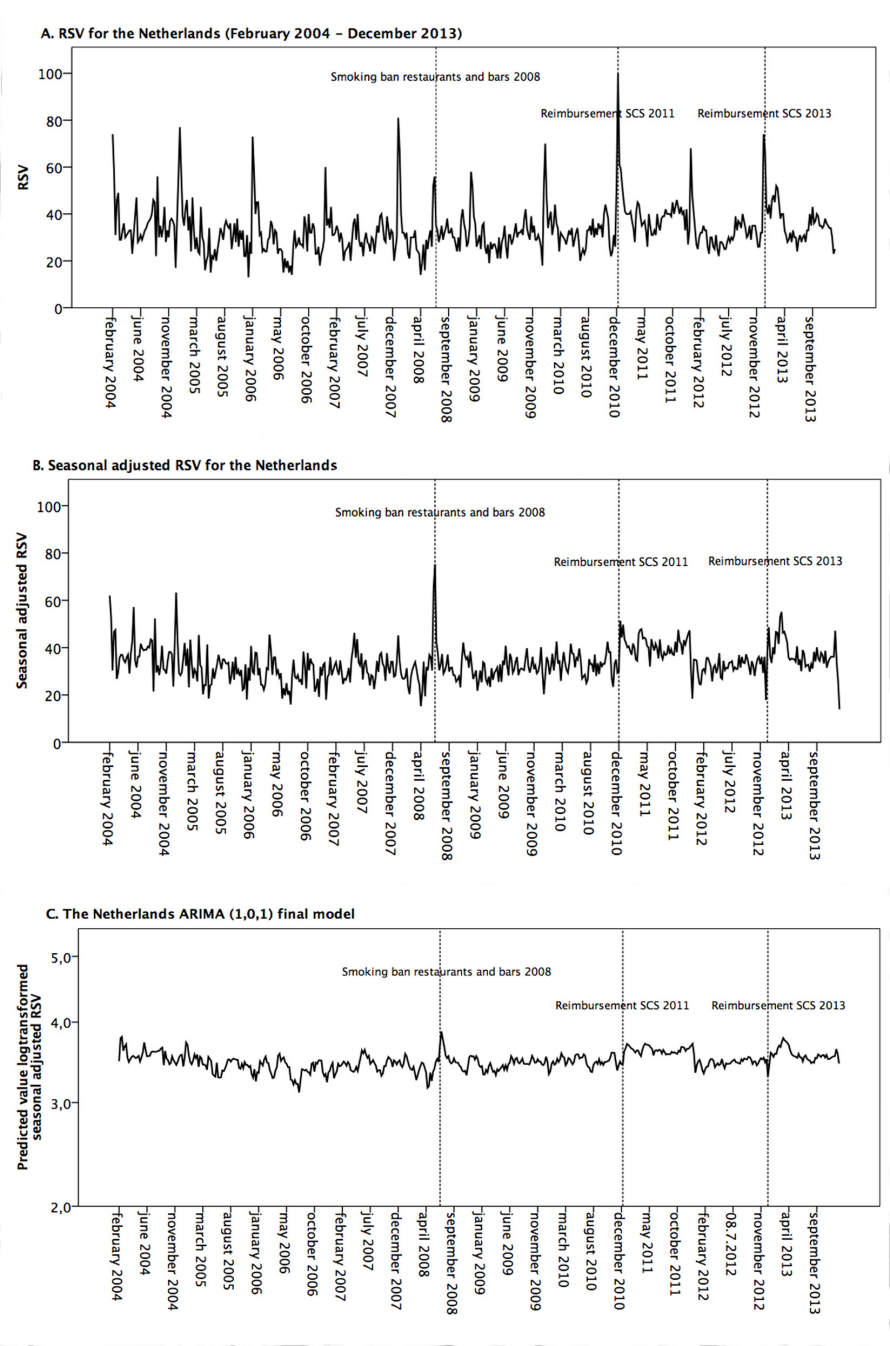
Google Trends RSV data of ‘quit smoking’ for the Netherlands. Dutch RSV (A), Dutch seasonally adjusted RSV (B) and as ARIMA (1,0,1) model (C).

This study was conducted using a quasi-experimental design to examine trends in search queries around interventions. Interrupted time series analysis allows the comparison of outcome measures before and after the implementation of an intervention [29,30]. Therefore, this method can be used to analyse the effects of the introduction of new tobacco control policies by using RSV data. Time series analyses have been previously used to measure the impact of tobacco control policies on smoking prevalence in Australia [31]. RSV data were made available by Google Trends from 2004 onwards. Dutch RSV data for the query ‘quit smoking’ (‘stoppen met roken’) were retrieved for the 2004 to 2013 period on a weekly scale. Based on the availability of Google Trends data, three Dutch smoking cessation interventions could be included: the smoking ban of 2008, the reimbursement of SCS in 2011 and the reintroduction of the reimbursement of 2013 (Table 1). In January 2004, a national smoke-free legislation at the workplace was implemented. Since no pre-intervention data were available, the effect of this legislation could not be analysed. Therefore, January 2004 was excluded from the analysis. In total, data from 517 weekly time points were retrieved, with the highest RSV observed in the middle of 2008 (Fig 1). Sensitivity analysis showed that our results were robust with exclusion of the first 6 months of 2004 (instead of January only).

**Table 1:** Tobacco control policies implemented in the Netherlands and Belgium in the period 2004–2013.

Starting date	Country	Tobacco control policy
01-01-2005	Netherlands	Smoking ban hospitals and nursing homes
01-01-2007	Belgium	Smoking ban restaurants (small establishments excluded)
07-01-2007	Belgium	Smoking ban diners at public places (shopping malls, sports centers)
07-01-2008	Netherlands	Smoking ban bars and restaurants
01-01-2010	Belgium	Smoking ban food services places (small establishments excluded)
01-01-2011	Netherlands	Reimbursement SCS costs (duration 1 year)
30-06-2011	Belgium	Smoking ban all restaurants and snack points
01-01-2013	Netherlands	Restart Reimbursement SCS costs

**Fig 2:**
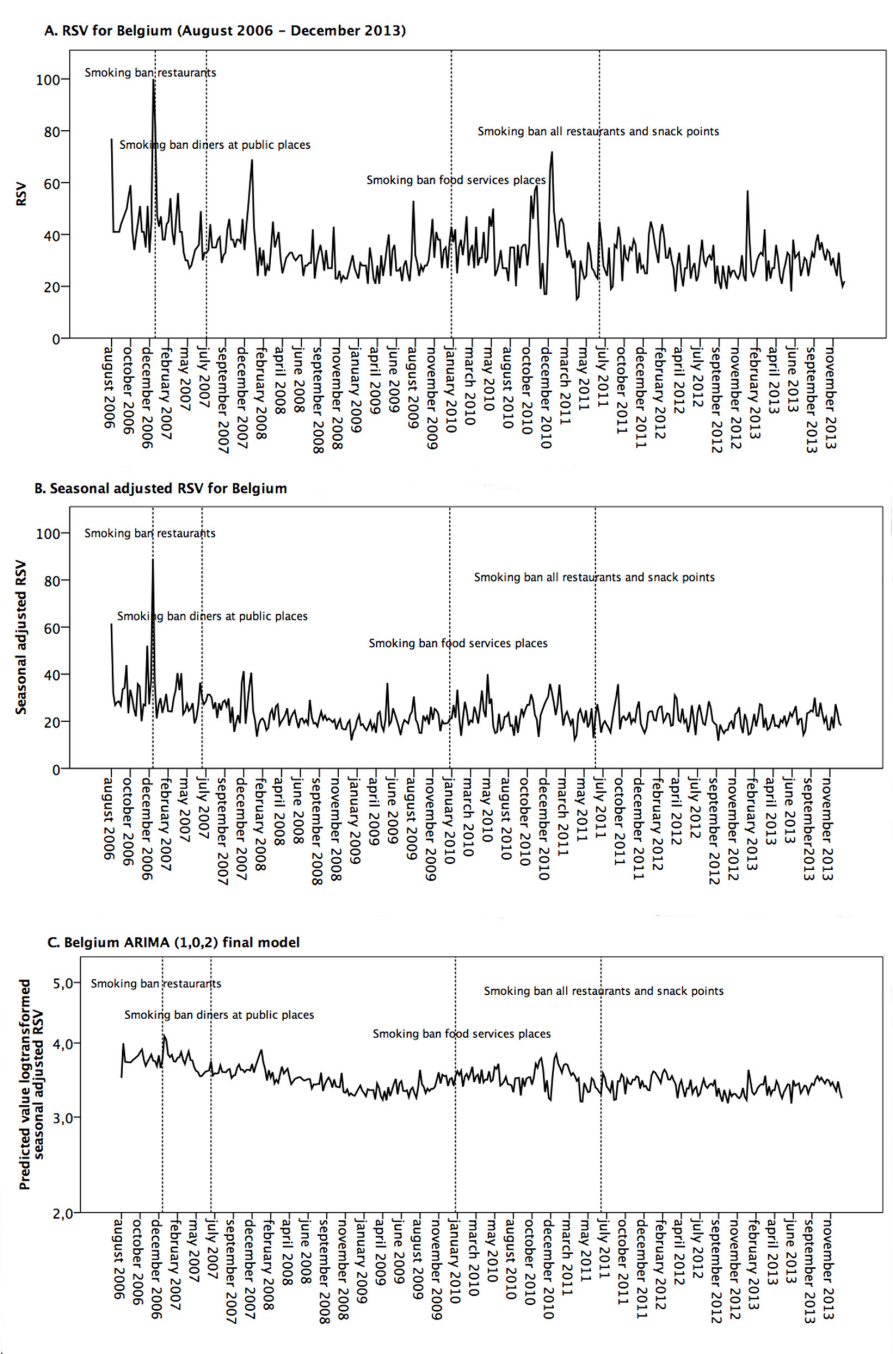
Google Trends RSV data of ‘quit smoking’ for Belgium. Belgian RSV (A), Belgian RSV seasonally adjusted (B) and as ARIMA (1,0,2) Model (C).

We added the Dutch speaking part of Belgium as the control group, since Belgium is the country that is most comparable to the Netherlands in terms of geography, language, history and culture. Moreover, Belgium and the Netherlands have a similar smoking prevalence [18]. We used the same Dutch term on ‘quit smoking’ to obtain Belgian RSV data. The Belgian RSV ‘quit smoking’ data comprised a large number of missing data points until mid-2006. Therefore the period of January 2004 to August 2006 was excluded from the Belgian analysis. In total, data from 387 weekly time points were retrieved, with the highest RSV observed in the beginning of 2007 (Fig 2).

### Statistical analysis

To correct for seasonality, a seasonal decomposition in SPSS (version 21) was performed and the seasonally adjusted series were used for the analyses [32]. This enabled us to adjust for the peak in search queries around, among others, the first day of January when people make New Year’s resolutions to quit smoking. The seasonal adjusted series were log transformed in order to stabilize variance over time [33].

An interrupted time series analysis was performed on the adjusted data [29,30]. Autoregressive integrated moving average (ARIMA) modelling was used to account for dependency between data points in time series. ARIMA modelling is able to account for non-independence between the data points in the time series, which would otherwise cause standard errors to be estimated incorrectly. When using ARIMA modelling, the length between measured time periods should be constant. Furthermore, a substantial amount of data points is necessary. Time series analysis using ARIMA modelling is accomplished in three phases: identification, estimation and diagnosis. In the identification phase ACF (autocorrelation function) and PACF (partial autocorrelation function) plots were examined to see which patterns are present in the data. The ACF plot is a bar chart of the coefficients of correlation between a time series and lags of itself. The PACF plot is a bar chart of the correlation coefficients between the series and lags of itself that are not explained by correlation at all lower order lags. Based on the visual inspection of the ACF and PACF, initial autoregressive (AR) and moving average (MA) terms were determined as a tentative model. In the estimation phase the tentative model was fitted to the seasonally adjusted RSV series to determine the fit of the model. Extra AR and MA terms were added to make sure no terms were left out of the model. In the diagnosis phase the best fitting model was determined based on residual ACF and PACF and the final model was used for further analyses [33,34].

The models were used to estimate the association with various tobacco control policies implemented in the Netherlands and Belgium (Table 1). Based on findings in literature, effects were estimated to be relatively short lived, with an increase in search queries before and shortly after the implementation of the legislation and a decrease in search queries a few weeks or months [15–17] afterwards. To assess the timing and duration of the effect of the tobacco control policies, twelve intervention effect periods were distinguished, ranging in duration from sixteen weeks up to one week before the intervention and one week up to 52 weeks afterwards. This method was chosen to minimize the amount of effect periods, in order to avoid the risks of multiple testing. Moreover, a similar approach is used in an article by Szatkowski et al. [15]. For each of the tobacco control policies, the respective periods were modeled as binary intervention dummies coded ‘1’ for the duration of the intervention period and coded ‘0’ for the period before and after that period of intervention. To clarify, for the effect period of one week after the intervention, this specific intervention variable was only coded as ‘1’ for the first week after the intervention. All other weeks were coded as ‘0’. Similarly, for the effect period of 5 to 8 weeks after the intervention, a variable was added in which week 5, 6, 7 and 8 were coded as ‘1’ and all other weeks were coded as ‘0’.

The dummies for the different periods were added simultaneously to the final model to estimate the intervention effects according to each period [35]. Output was measured in percentage points relative to the expected level without the influence of the tobacco control policies. This process was repeated for each of the Dutch tobacco control policies.

The Belgian ARIMA analysis was used as a control. The same regression model was applied to the Belgian model to study whether the changes observed around the Dutch interventions would be restricted to the Netherlands or not. Dummy variables related to Belgian policies (Table 1) were also added to the Belgian model to control for the effect of these Belgian interventions. Effect sizes and confidence intervals were estimated and a p-value smaller than 0.05 was considered to imply statistical significance. All analyses were performed using SPSS 21.

## Results

The appropriate ARIMA model of Dutch data was determined as a mixed autoregressive moving average model with one autoregressive lag and one moving average lag (ARIMA (1,0,1)) (Fig 1) and the Belgian model was determined as a mixed autoregressive moving average model with one autoregressive lag and two moving average lags (ARIMA (1,0,2)) model (Fig 2).

Four Belgian tobacco control policies were added to the Belgian ARIMA model to measure the effects of Belgian tobacco control policies on smoking cessation-related behaviour (Table 2). The effects of the Belgian tobacco control policies were relatively weak. The smoking ban for diners in public places of July 2007 and the smoking ban in most food services places of January 2010 were not associated with any increases in relative search volume. The policies that were sometimes associated with increases in Belgian RSV were the partial smoking ban in restaurants in 2007 and the smoking ban in all restaurants and snack points in 2011. However, we found only a few significant effects (possibly resulting from multiple testing) and no substantial effect sizes. Therefore, the intervention variables of these two policies were added to the Belgian ARIMA (1,0,2) control model.

**Table 2:** ARIMA modelling outcomes for Belgian smoking cessation policies of Belgian RSV data.

	Period	Estimated ratio of change in RSV	95% CI
**Smoking ban restaurants 2007 (small establishments excluded)**			
*Weeks before intervention*	9–16	1.07	(0.95–1.20)
	5–8	0.99	(0.85–1.16)
	3–4	1.00	(0.84–1.21)
	2	1.14	(0.92–1.40)
	1	0.93	(0.75–1.15)
*Weeks after intervention*	1	1.11	(0.89–1.37)
	2	1.42*	(1.14–1.75)
	3–4	1.11	(0.92–1.35)
	5–8	1.05	(0.88–1.25)
	9–16	1.05	(0.89–1.24)
	17–32	1.01	(0.87–1.17)
	33–52	0.98	(0.88–1.10)
**Smoking ban diners at public places 2007**			
*Weeks before intervention*	9–16	1.02	(0.89–1.15)
	5–8	1.05	(0.90–1.22)
	3–4	1.00	(0.83–1.20)
	2	1.00	(0.81–1.24)
	1	1.07	(0.86–1.33)
*Weeks after intervention*	1	1.03	(0.83–1.27)
	2	1.09	(0.88–1.35)
	3–4	0.97	(0.80–1.18)
	5–8	1.04	(0.87–1.24)
	9–16	0.97	(0.83–1.14)
	17–32	0.93	(0.81–1.08)
	33–52	0.99	(0.88–1.11)
**Smoking ban food services places (small establishments excluded)**			
*Weeks before intervention*	9–16	0.95	(0.85–1.07)
	5–8	1.00	(0.86–1.17)
	3–4	1.02	(0.85–1.23)
	2	1.07	(0.86–1.31)
	1	1.04	(0.84–1.29)
*Weeks after intervention*	1	0.99	(0.80–1.23)
	2	1.04	(0.84–1.29)
	3–4	0.96	(0.79–1.17)
	5–8	1.02	(0.85–1.21)
	9–16	1.00	(0.85–1.17)
	17–32	1.01	(0.87–1.17)
	33–52	0.96	(0.86–1.08)
**Smoking ban all restaurants and snack points**			
*Weeks before intervention*	9–16	0.90	(0.80–1.01)
	5–8	0.95	(0.81–1.10)
	3–4	1.01	(0.84–1.22)
	2	0.94	(0.73–1.15)
	1	0.97	(0.76–1.16)
*Weeks after intervention*	1	0.89	(0.72–1.10)
	2	0.90	(0.73–1.12)
	3–4	1.14	(0.94–1.38)
	5–8	1.00	(0.84–1.19)
	9–16	0.95	(0.81–1.11)
	17–32	0.96	(0.84–1.11)
	33–52	0.99	(0.88–1.11)

CI: confidence interval

* Significant at P<0.05.

**Fig 3:**
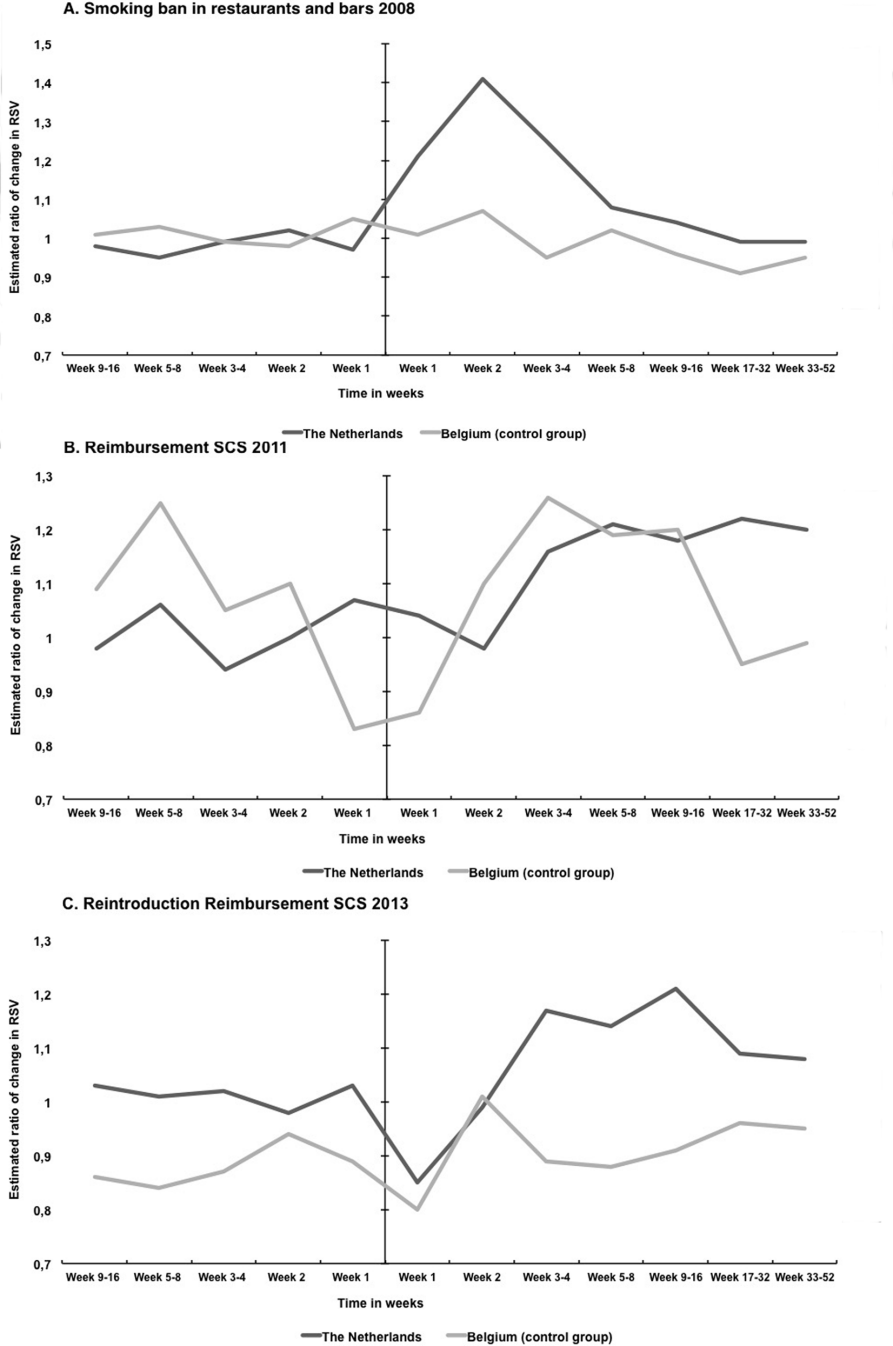
Proportional change in Google Trends RSV of ‘quit smoking’ before and after the introduction of three Dutch tobacco control interventions in the Netherlands and Belgium. Interventions are the smoking ban in restaurants and bars of 2008 (A), the reimbursement of SCS in 2011 (B) and the reimbursement of SCS in 2013 (C).

Table 3 and Fig 3 show the estimated changes in RSV ratio around three tobacco control policies implemented in the Netherlands. Increases in RSV were estimated as compared to the estimates from a model without the occurrence of any interventions. Before the start of the smoking ban in 2008 there appears to be no increase in RSV. For the smoking ban of 2008, statistically significant increases in RSV were found for one week (21%, 95% CI 4–42), two weeks (41%, 95% CI 21–64) and three to four weeks (41%, 95% CI 8–43) after the introduction of the policy. After four weeks, the increase in RSV became non-significant and after 16 weeks the RSV returned to the level estimated without the intervention. In the control group of Belgian RSV data, no significant changes were found.

**Table 3:** ARIMA modelling outcomes for three Dutch smoking cessation policies of RSV data from the Netherlands and Belgium (corrected for Belgian interventions).

		The Netherlands		Belgium (control group)	
	Period	Estimated ratio of change in RSV	95% CI	Estimated ratio of change in RSV	95% CI
**Smoking ban restaurants and bars 2008**					
*Weeks before intervention*	9–16	0.98	(0.89–1.07)	1.01	(0.90–1.12)
	5–8	0.95	(0.84–1.07)	1.03	(0.91–1.18)
	3–4	0.99	(0.86–1.13)	0.99	(0.84–1.15)
	2	1.02	(0.87–1.18)	0.98	(0.82–1.19)
	1	0.97	(0.83–1.14)	1.05	(0.87–1.27)
*Weeks after intervention*	1	1.21*	(1.04–1.42)	1.01	(0.84–1.22)
	2	1.41*	(1.21–1.64)	1.07	(0.89–1.29)
	3–4	1.25*	(1.08–1.43)	0.95	(0.81–1.13)
	5–8	1.08	(0.96–1.22)	1.02	(0.88–1.18)
	9–16	1.04	(0.94–1.15)	0.96	(0.84–1.10)
	17–32	0.99	(0.91–1.08)	0.91	(0.81–1.02)
	33–52	0.99	(0.92–1.07)	0.95	(0.86–1.05)
**Reimbursement SCS 2011**					
*Weeks before intervention*	9–16	0.98	(0.89–1.07)	1.09	(0.98–1.20)
	5–8	1.06	(0.94–1.20)	1.25*	(1.09–1.43)
	3–4	0.94	(0.82–1.08)	1.05	(0.89–1.22)
	2	1.00	(0.86–1.16)	1.10	(0.92–1.32)
	1	1.07	(0.89–1.21)	0.83	(0.69–1.00)
*Weeks after intervention*	1	1.04	(0.85–1.17)	0.86	(0.71–1.04)
	2	0.98	(0.84–1.14)	1.10	(0.91–1.32)
	3–4	1.16*	(1.01–1.33)	1.26*	(1.07–1.48)
	5–8	1.21*	(1.08–1.37)	1.19*	(1.03–1.38)
	9–16	1.18*	(1.06–1.30)	1.20*	(1.04–1.35)
	17–32	1.22*	(1.13–1.33)	0.95	(0.81–1.10)
	33–52	1.20*	(1.13–1.33)	0.99	(0.89–1.09)
**Reimbursement SCS 2013**					
*Weeks before intervention*	9–16	1.03	(0.94–1.13)	0.86*	(0.76–0.97)
	5–8	1.01	(0.90–1.14)	0.84*	(0.70–0.98)
	3–4	1.02	(0.89–1.17)	0.87	(0.70–1.04)
	2	0.98	(0.84–1.14)	0.94	(0.75–1.14)
	1	1.03	(0.88–1.21)	0.89	(0.69–1.09)
*Weeks after intervention*	1	0.85*	(0.72–0.99)	0.80*	(0.66–0.98)
	2	0.99	(0.85–1.15)	1.01	(0.82–1.23)
	3–4	1.17*	(1.02–1.34)	0.89	(0.74–1.27)
	5–8	1.14*	(1.01–1.29)	0.88	(0.74–1.05)
	9–16	1.21*	(1.09–1.33)	0.91	(0.76–1.08)
	17–32	1.09*	(1.00–1.19)	0.96	(0.79–1.16)
	33–52	1.08	(1.00–1.18)	0.95	(0.77–1.17)

CI: confidence interval

* Significant at P<0.05.

The introduction of the reimbursement of SCS in 2011 was associated with a significant increase in RSV after three to four weeks (16%, 95% CI 1–33), five to eight weeks (21%, 95% CI: 8–37), nine to 16 weeks (18%, 95% CI: 6–30), 17 to 32 weeks (22%, 95% CI: 13–33) and 33 to 52 weeks (20%, 95% CI: 13–23) after the intervention. For three of these periods in 2011, a statistically significant increase was also found in the Belgian RSV. In addition, a significant increase in Belgian RVS occurred 5–8 weeks before the introduction.

The observed effect of the reimbursement of SCS in 2013 was shorter and had a smaller magnitude compared to the reimbursement of SCS in 2011. The reintroduction of the reimbursement of SCS in 2013 was associated with a statistically significant increase in RSV for three to four weeks (17%, 95% CI 2–34), five to eight weeks (14%, 95% CI 1–29), nine to 16 weeks (21%, 95% CI 9–33) and 17 to 32 weeks (9%, 95% CI 0–19) after the intervention. In the control group, significant decreases in RSV occurred before the start and one week after the intervention. There was no increase of RSV in the control group after the Dutch reimbursement of SCS in 2013 was implemented.

## Discussion

The aim of this study was to assess the magnitude and duration of the impact of the Dutch smoke-free legislation and reimbursement of smoking cessation support on internet search query data. The smoke-free legislation in bars and restaurants was associated with a short-term increase in RSV, compared to the almost constant Belgian trend in this period. We found that the reimbursement of SCS in 2011 was associated with a significant increase in RSV for three weeks to one year after the start of the intervention and the reintroduction of the reimbursement of SCS in 2013 was followed by an increase in RSV for three to 32 weeks after the start of the intervention. For the control group, significant increases were found for the reimbursement of SCS in 2011, but no increase was found for the reimbursement of SCS in 2013.

### Evaluation of methodology

ARIMA interrupted time series modelling is appropriate to evaluate several types of community level health interventions [36]. ARIMA modelling is especially suitable when using detailed time series data such as Google Trends data [37]. Our study shows that Google data, measured in RSV, could be a useful tool to measure the effect of tobacco control policies, based on its ability to represent large populations. This is in agreement with Cavazos-Rehg et al. [23] who state that the results of their study validate the potential of Google trends as a valuable monitoring tool for tobacco use. However, RSV is a proxy and therefore does not give information on actual smoking cessation behaviour.

Time series analyses that distinguish multiple exposure periods are susceptible to the risk of multiple testing. However, we think that the results of this study are not influenced by multiple testing, since statistically significant results were found in far more than five per cent of the exposure periods. Another concern with time series is the statistical power. In time series, statistical power is primarily affected by the amount of post and pre intervention data points [38]. In our study, a total of 517 weekly data points of the Netherlands and 387 data points of Belgium were available, which seem sufficient for our purposes. According to Thomas [39], a method to estimate power retrospectively is to look at the width of confidence intervals While large effects could be demonstrated, we may not have been able to demonstrate smaller effects with statistical significance. For example, in the case of the introduction of the smoking ban in 2008, we could demonstrate changes occurring within 1 to 4 weeks after the intervention, but smaller changes in the same direction at the longer term could not be demonstrated with statistical significance, possibly due to inadequate power.

One of the main concerns of using internet search query data is the representativeness of such data. The prevalence of internet use is higher among individuals who are younger, more educated and have a higher income [40]. Moreover, compared to older people, those in their twenties may be more likely to think about quitting in response to smoking bans in bars and restaurants [11]. Therefore, our results may not be representative for the entire Dutch population. However, it has recently been demonstrated that individuals of sixty years and older are equally likely to use internet search engines to search for health related information compared to adolescents [26]. Furthermore, in the Netherlands and in Belgium, the percentage of the population having internet access is very high, 95% and 80% respectively [41].

Out of the seven Dutch and Belgian tobacco control policies used in our analyses, four were implemented at the first of January. Since they coincided with the New Year’s resolutions peak, it was difficult to separate the impact of the policies from this peak. We corrected for this by performing a seasonality correction, which eliminated the average peak found at the first week of January. However, this was not a completely accurate process, since Google Trends provides weekly data from Monday till Sunday and each subsequent year started on a different weekday. Because of the potential of residual confounding, results found in the first week of January should be interpreted with caution.

After the implementation of the reimbursement of SCS in 2011, an increase in RSV was found not only in the Netherlands, but also in Belgium. We could speculate that the increase in RSV could be caused by Dutch speaking media influencing not only the Dutch, but also Belgian residents. However, after the implementation of the reimbursement of SCS in 2013 no effects on Belgian RSV were found. A possible explanation might be that the introduction of the Dutch reimbursement policy of 2011 was accompanied by a media campaign [42], as opposed to the reimbursement policies of 2013. This could have influenced the exposure of Belgian residents towards this policy. Nonetheless, the Belgian results show that despite our time series study design, mid-term effects such as observed in relationship to the Dutch reimbursement policies of 2011 and 2013, may be subject to residual confounding, since more information is necessary to correct for media exposure.

### Substantive interpretations

The findings of this study are consistent with other types of studies on the effect of tobacco control legislation on quit attempts and cessation rates. Earlier research on the Dutch smoke-free legislation in the hospitality industry found a temporary increase in quit attempts and a temporary decrease in smoking prevalence [10,11]. Bar visitors were more likely to quit smoking after the implementation of the smoking ban compared to the period before the implementation [10]. In New Zealand, a large increase in quit line telephone call registrations was found in the six-month period after the implementation of a smoke free legislation [12,13]. In England, a significant increase in quit attempts was found for two months after the start of smoke-free legislation [14]. This suggests that the effect of the smoking ban on smoking cessation related behaviour is short to mid-term, which is comparable with the short-term effect that was found in this study. The short duration and large magnitude of increase in research queries is suggestive of a “shock effect”. This would imply that the effect weakens quickly when the population adjusts to the new situation [43,44].

A Dutch study found that the implementation of SCS led to a decrease in smoking prevalence in the six months afterwards, thus suggesting long-term effects on smoking prevalence [14]. Also, a randomized controlled trial on free offer of nicotine patches through telephonic smoking quit lines found an increase in quit attempts and a decrease in smoking prevalence when the period of offering free nicotine patches increased [45]. A study in Massachusetts found that reimbursement of SCS was followed by a significant decline in smoking prevalence for at least two years with a yearly relative decline of 15,2% [46].

The available evidence thus suggests that the reimbursement of SCS had an effect on quit attempts and smoking prevalence for at least one year. This is consistent with our results of the reimbursement of SCS in 2011, but not with 2013. The shortened duration of the effects of the SCS policy in 2013 comparison with the SCS policy in 2011 may have been due to the fact that the same type of policies was repeated within a short time period. Moreover, a media campaign accompanied the introduction of the policy in 2011 but not in 2013 [46].

In contrast with the Dutch tobacco control policies, the Belgian tobacco control policies were not clearly associated with increases in Belgian RSV. This might be due to the complex history of tobacco control policies in Belgium, where new smoke-free policies were introduced with multiple exceptions and a limited enforcement [47].

### Conclusion and implications

Our results provide some empirical support to believe that the introduction of a smoking ban in restaurant and bars is associated with a short-term increase in online searching for information about smoking cessation. The reimbursement of SCS costs seems to have a mid to longer-term effect on searching behaviour. This evidence is consistent with earlier results of previous studies that report a positive impact of such policies in trends on smoking cessation rates and specifies with more accuracy the magnitude and duration of these effects.

Information on the time frame during which tobacco control policies may affect smoking cessation behaviour and its precursors, can be used to improve the implementation of these policies. Periods when smokers are more likely to contemplate smoking cessation may provide windows of opportunity for interventions targeted at smokers who contemplate smoking.

## References

[1] Danaei G, Ding EL, Mozaffarian D, Taylor B, Rehm J, Murray CJL, et al The preventable causes of death in the United States: comparative risk assessment of dietary, lifestyle, and metabolic risk factors. PLoS Med. 2009;6: e1000058 10.1371/journal.pmed.1000058 19399161 PMC2667673

[2] STIVORO. Kerncijfers roken in Nederland 2012. Een overzicht van recente Nederlandse basisgegevens over rookgedrag. Den Haag; 2013.

[3] Blanke DD, Da Costa V. Tools for advancing tobacco control in the 21st century. Tobacco control legislation: an introductory guide. Geneva, Switzerland; 2004.

[4] Ministry of Health WaS. Tobacco Act. 1990.

[5] Nagelhout GE, Levy DT, Blackman K, Currie L, Clancy L, Willemsen MC. The effect of tobacco control policies on smoking prevalence and smoking-attributable deaths. Findings from the Netherlands SimSmoke Tobacco Control Policy Simulation Model. Addiction. 2012;107: 407–16. 10.1111/j.1360-0443.2011.03642.x 21906197

[6] Nagelhout GE, Crone MR, van den Putte B, Willemsen MC, Fong GT, de Vries H. Age and educational inequalities in smoking cessation due to three population-level tobacco control interventions: findings from the International Tobacco Control (ITC) Netherlands Survey. Health Educ Res. 2013;28: 83–91. 10.1093/her/cys101 23087009

[7] Nagelhout GE, de Vries H, Fong GT, Candel MJJM, Thrasher JF, van den Putte B, et al Pathways of change explaining the effect of smoke-free legislation on smoking cessation in The Netherlands. An application of the international tobacco control conceptual model. Nicotine Tob Res. 2012;14: 1474–82. 10.1093/ntr/nts081 22491892 PMC3509014

[8] Thyrian JR, Panagiotakos DB, Polychronopoulos E, West R, Zatonski W, John U. The relationship between smokers’ motivation to quit and intensity of tobacco control at the population level: a comparison of five European countries. BMC Public Health. 2008;8 10.1186/1471-2458-8-2 PMC2245926 18173845

[9] Warner KE, Mendez D. Tobacco control policy in developed countries: yesterday, today, and tomorrow. Nicotine Tob Res. 2010;12: 876–87. 10.1093/ntr/ntq125 20702814

[10] Nagelhout GE, Willemsen MC, de Vries H. The population impact of smoke-free workplace and hospitality industry legislation on smoking behaviour. Findings from a national population survey. Addiction. 2011;106: 816–23. 10.1111/j.1360-0443.2010.03247.x 21182553

[11] Nagelhout GE, de Vries H, Boudreau C, Allwright S, McNeill A, van den Putte B, et al Comparative impact of smoke-free legislation on smoking cessation in three European countries. Eur J Public Health. 2012;22 Suppl 1: 4–9. 10.1093/eurpub/ckr203 22294778 PMC3451298

[12] Edwards R, Thomson G, Wilson N, Waa A, Bullen C, O’Dea D, et al After the smoke has cleared: evaluation of the impact of a new national smoke-free law in New Zealand. Tob Control. 2008;17: e2 10.1136/tc.2007.020347 18218788

[13] Wilson N, Sertsou G, Edwards R, Thomson G, Grigg M, Li J. A new national smokefree law increased calls to a national quitline. BMC Public Health. 2007;7: 75 10.1186/1471-2458-7-75 17488525 PMC1871580

[14] Hackshaw L, McEwen A, West R, Bauld L. Quit attempts in response to smoke-free legislation in England. Tob Control. 2010;19: 160–4. 10.1136/tc.2009.032656 20378592

[15] Szatkowski L, Coleman T, McNeill A, Lewis S. The impact of the introduction of smoke-free legislation on prescribing of stop-smoking medications in England. Addiction. 2011;106: 1827–34. 10.1111/j.1360-0443.2011.03494.x 21561500

[16] Albers AB, Siegel M, Cheng DM, Biener L, Rigotti NA. Effect of smoking regulations in local restaurants on smokers’ anti-smoking attitudes and quitting behaviours. Tob Control. 2007;16: 101–6. 10.1136/tc.2006.017426 17400947 PMC2598469

[17] Wakefield MA. Effect of restrictions on smoking at home, at school, and in public places on teenage smoking: cross sectional study. BMJ. 2000;321: 333–337. 10.1136/bmj.321.7257.333 10926588 PMC27448

[18] Joossens L, Raw M. The Tobacco Control Scale in Europe. Brussels, Belgium; 2014.

[19] Carneiro HA, Mylonakis E. Google trends: a web-based tool for real-time surveillance of disease outbreaks. Clin Infect Dis. 2009;49: 1557–64. 10.1086/630200 19845471

[20] Ayers JW, Althouse BM, Allem J-P, Childers MA, Zafar W, Latkin C, et al Novel surveillance of psychological distress during the great recession. J Affect Disord. 2012;142: 323–30. 10.1016/j.jad.2012.05.005 22835843 PMC4670615

[21] Yang AC, Huang NE, Peng C-K, Tsai S-J. Do seasons have an influence on the incidence of depression? The use of an internet search engine query data as a proxy of human affect. Neylon C, editor. PLoS One. Public Library of Science; 2010;5: e13728 10.1371/journal.pone.0013728 21060851 PMC2965678

[22] Jo CL, Ayers JW, Althouse BM, Emery S, Huang J, Ribisl KM. US consumer interest in non-cigarette tobacco products spikes around the 2009 federal tobacco tax increase. Tob Control. 2014; Published online first: tobaccocontrol–2013–051261. 10.1136/tobaccocontrol-2013-051261 PMC4122659 24500270

[23] Cavazos-Rehg PA, Krauss MJ, Spitznagel EL, Lowery A, Grucza RA, Chaloupka FJ, et al Monitoring of non-cigarette tobacco use using Google Trends. Tob Control. 2014; Published online first: tobaccocontrol–2013–051276. 10.1136/tobaccocontrol-2013-051276 PMC4122644 24500269

[24] Ayers JW, Ribisl KM, Brownstein JS. Tracking the rise in popularity of electronic nicotine delivery systems (electronic cigarettes) using search query surveillance. Am J Prev Med. Elsevier; 2011;40: 448–53. 10.1016/j.amepre.2010.12.007 21406279

[25] Ayers JW, Ribisl K, Brownstein JS. Using search query surveillance to monitor tax avoidance and smoking cessation following the United States’ 2009 “SCHIP” cigarette tax increase. Carr J, editor. PLoS One. Public Library of Science; 2011;6: e16777 10.1371/journal.pone.0016777 21436883 PMC3059206

[26] Ayers JW, Althouse BM, Ribisl KM, Emery S. Digital Detection for Tobacco Control: Online Reactions to the United States’ 2009 Cigarette Excise Tax Increase. Nicotine Tob Res. 2013;16: 576–83. 10.1093/ntr/ntt186 24323570 PMC3977484

[27] Google Support. Trends Help [Internet]. [cited 6 Mar 2014]. Available: https://support.google.com/trends/?hl=en#

[28] Seifter A, Schwarzwalder A, Geis K, Aucott J. The utility of “Google Trends” for epidemiological research: Lyme disease as an example. Geospat Health. 2010;4: 135–137. 20503183 10.4081/gh.2010.195

[29] Cook TD, Campbell DT. Quasi-experimentation: design & analysis issues for field settings 3rd ed. Rand McNally College Pub. Co.; 1979.

[30] Shadish WR, Cook TD. The renaissance of field experimentation in evaluating interventions. Annu Rev Psychol. Annual Reviews; 2009;60: 607–29. 10.1146/annurev.psych.60.110707.163544 18616391

[31] Wakefield MA, Coomber K, Durkin SJ, Scollo M, Bayly M, Spittal MJ, et al Time series analysis of the impact of tobacco control policies on smoking prevalence among Australian adults, 2001–2011. Bull World Health Organ. World Health Organization; 2014;92: 413–22. 10.2471/BLT.13.118448 24940015 PMC4047797

[32] IBM Corporation. SPSS. 2012.

[33] Tabachnick BG, Fidell LS. Using multivariate statistics 4th ed. University of Michigan: Allyn and Bacon; 2001.

[34] Nelson BK. Time Series Analysis Using Autoregressive Integrated Moving Average (ARIMA) Models. Acad Emerg Med. 1998;5: 739–744. 10.1111/j.1553-2712.1998.tb02493.x 9678399

[35] Hanbury A, Farley K, Thompson C, Wilson PM, Chambers D, Holmes H. Immediate versus sustained effects: interrupted time series analysis of a tailored intervention. Implement Sci. 2013;8: 130 10.1186/1748-5908-8-130 24188718 PMC4228338

[36] Chandran A, Pérez-Núñez R, Bachani AM, Híjar M, Salinas-Rodríguez A, Hyder AA. Early impact of a national multi-faceted road safety intervention program in Mexico: results of a time-series analysis. PLoS One. 2014;9: e87482 10.1371/journal.pone.0087482 24498114 PMC3909119

[37] Zhang X, Zhang T, Young AA, Li X. Applications and comparisons of four time series models in epidemiological surveillance data. PLoS One. 2014;9: e88075 10.1371/journal.pone.0088075 24505382 PMC3914930

[38] Zhang F, Wagner AK, Ross-Degnan D. Simulation-based power calculation for designing interrupted time series analyses of health policy interventions. J Clin Epidemiol. 2011;64: 1252–61. 10.1016/j.jclinepi.2011.02.007 21640554

[39] Thomas L. Retrospective Power Analysis. Conserv Biol. 1997;11: 276–280. 10.1046/j.1523-1739.1997.96102.x

[40] PEW Research Center’s Internet & American Life Project. Internet User Demographics [Internet]. 2014 [cited 26 May 2014]. Available: http://www.pewinternet.org/data-trend/internet-use/latest-stats/

[41] European Union. Eurostat: Database [Internet]. 2015. Available: http://ec.europa.eu/eurostat/data/database

[42] Willems RA, Willemsen MC, Nagelhout GE, Smit ES, Janssen E, van den Putte B, et al. Evaluatie van de “Echt stoppen met roken kan met de juiste hulp” campagne. Maastricht; 2012.

[43] Hu TW, Sung HY, Keeler TE. Reducing cigarette consumption in California: tobacco taxes vs an anti-smoking media campaign. Am J Public Health. American Public Health Association; 1995;85: 1218–1222. 10.2105/AJPH.85.9.1218 7661228 PMC1615589

[44] Pekurinen M, Valtonen H. Price, policy and consumption of tobacco: The Finnish experience. Soc Sci Med. 1987;25: 875–881. 10.1016/0277-9536(87)90256-5 3686116

[45] McAfee TA, Bush T, Deprey TM, Mahoney LD, Zbikowski SM, Fellows JL, et al Nicotine patches and uninsured quitline callers. A randomized trial of two versus eight weeks. Am J Prev Med. 2008;35: 103–10. 10.1016/j.amepre.2008.04.017 18617079

[46] Nagelhout GE, Willemsen MC, van den Putte B, de Vries H, Willems RA, Segaar D. Effectiveness of a national reimbursement policy and accompanying media attention on use of cessation treatment and on smoking cessation: a real-world study in the Netherlands. Tob Control. 2014; tobaccocontrol–2013–051430–. 10.1136/tobaccocontrol-2013-051430 24842854

[47] Joossens L, Raw M. Progress in Tobacco Control in 30 European Countries from 2005 to 2007. Basel, Switserland; 2007.

